# 

**Published:** 1908-08-08

**Authors:** 


### BOOKS RECEIVED.

The Caxton Publishing Co.

"The Edinburgh Stereoscopic Atlas of Obstetrics." Series II.

Cornish Bros., Birmingham.

"Points of Practice in Maladies of the H[illegible]art." By Sir James Sawyer, M.D.

J. B. Lippincott Co.

"International Clinics." 18th series. Vol. II.

"Religion and Medicine." By S. McComb, M.A., D.D., Elwood Worcester, D.D., Ph.D., and Isador H. Coreat, M.D.

W. B. Saunders and Co.

"Practice of Medicine for Nurses." By G. H. Hoxie, M.D.

Simpkin, Marshall and Co.

"The Cure of 'Bad Throats' by Good Breathing." By Eric Robertson, M.A.

THE HOSPITAL August 8, 1908.
*Name* ..........................................................................................
*Address* ......................................................................................
..................................................................................................
This Coupon must accompany manuscript or contributions intended for The Hospital.

